# Successes and challenges in optimizing the viral load cascade to improve antiretroviral therapy adherence and rationalize second‐line switches in Swaziland

**DOI:** 10.1002/jia2.25194

**Published:** 2018-10-22

**Authors:** David Etoori, Iza Ciglenecki, Mpumelelo Ndlangamandla, Celeste G Edwards, Kiran Jobanputra, Munyaradzi Pasipamire, Gugu Maphalala, Chunfu Yang, Inoussa Zabsonre, Serge M Kabore, Javier Goiri, Roger Teck, Bernhard Kerschberger

**Affiliations:** ^1^ Research Department Médecins Sans Frontières Mbabane Swaziland; ^2^ Department of Population Health London School of Hygiene and Tropical Medicine London United Kingdom; ^3^ Research Department Médecins Sans Frontières Geneva Switzerland; ^4^ The Manson Unit Médecins Sans Frontières London United Kingdom; ^5^ Swaziland National AIDS Programme (SNAP) Ministry of Health Mbabane Swaziland; ^6^ Swaziland National Reference Laboratory (NRL) Ministry of Health Mbabane Swaziland; ^7^ Division of Global HIV/AIDS The Centre for Disease Control Atlanta GA USA; ^8^ South African Medical Unit Médecins Sans Frontières Cape Town South Africa

**Keywords:** viral load, ART, treatment monitoring, treatment switching, genotyping

## Abstract

**Introduction:**

As antiretroviral therapy (ART) is scaled up, more patients become eligible for routine viral load (VL) monitoring, the most important tool for monitoring ART efficacy. For HIV programmes to become effective, leakages along the VL cascade need to be minimized and treatment switching needs to be optimized. However, many HIV programmes in resource‐constrained settings report significant shortfalls.

**Methods:**

From a public sector HIV programme in rural Swaziland, we evaluated the VL cascade of adults (≥18 years) on ART from the time of the first elevated VL (>1000 copies/mL) between January 2013 and June 2014 to treatment switching by December 2015. We additionally described HIV drug resistance for patients with virological failure. We used descriptive statistics and Kaplan–Meier estimates to describe the different steps along the cascade and regression models to determine factors associated with outcomes.

**Results and Discussion:**

Of 828 patients with a first elevated VL, 252 (30.4%) did not receive any enhanced adherence counselling (EAC). Six hundred and ninety‐six (84.1%) patients had a follow‐up VL measurement, and the predictors of receiving a follow‐up VL were being a second‐line patient (adjusted hazard ratio (aHR): 0.72; *p *=* *0.051), Hlathikhulu health zone (aHR: 0.79; *p* = 0.013) and having received two EAC sessions (aHR: 1.31; *p *=* *0.023). Four hundred and ten patients (58.9%) achieved VL re‐suppression. Predictors of re‐suppression were age 50 to 64 (adjusted odds ratio (aOR): 2.02; *p *=* *0.015) compared with age 18 to 34 years, being on second‐line treatment (aOR: 3.29; *p *=* *0.003) and two (aOR: 1.66; *p *=* *0.045) or three (aOR: 1.86; *p *=* *0.003) EAC sessions. Of 278 patients eligible to switch to second‐line therapy, 120 (43.2%) had switched by the end of the study. Finally, of 155 successfully sequenced dried blood spots, 144 (92.9%) were from first‐line patients. Of these, 133 (positive predictive value: 92.4%) had resistance patterns that necessitated treatment switching.

**Conclusions:**

Patients on ART with high VLs were more likely to re‐suppress if they received EAC. Failure to re‐suppress after counselling was predictive of genotypically confirmed resistance patterns requiring treatment switching. Delays in switching were significant despite the ability of the WHO algorithm to predict treatment failure. Despite significant progress in recent years, enhanced focus on quality care along the VL cascade in resource‐limited settings is crucial.

## 1 Introduction

Routine viral load (VL) monitoring is the most important tool for assessing a patient's response to treatment, and assessing adherence to antiretroviral therapy (ART). The World Health Organization (WHO) recommends a VL test six and twelve months after starting ART and every twelve months thereafter 1, 2. For patients with elevated VLs (>1000 copies/mL), enhanced adherence counselling (EAC) is provided to identify adherence barriers. Virological failure is established if the next VL, taken between three and six months later, remains elevated 1. Clinicians should then switch treatment. Delaying treatment switching leads to accumulation of resistance mutations 3, 4, 5, unfavourable patient outcomes and increased risk of transmission of drug‐resistant strains 6, 7, 8, 9, 10, 11, 12, 13, 14, 15, 16, 17, 18, 19, 20, 21. If the second consecutive VL is re‐suppressed, patients usually remain on the same treatment regimen assuming that treatment adherence is restored without major drug resistance (DR).

Resource‐limited settings are scaling up routine VL monitoring. However, VL demonstration projects reported programmatic shortfalls and variability in quality of care along the VL cascade 22, 23. Other gaps include poorly equipped laboratories, lack of trained laboratory personnel, lack of up‐to‐date testing knowledge and blood sample transportation barriers 24.

Understanding of the gaps along the VL cascade remains limited but is needed to inform VL scale‐up in resource‐limited settings. We evaluated the performance of the VL cascade in a public health sector programme and describe the ability of the WHO VL testing algorithm to predict the need for treatment switching.

## 2 Methods

### 2.1 Setting

In Swaziland, 31% of the adult population (aged 18 to 49 years) are HIV positive 25, 26. Since 2007, the Swaziland Ministry of Health and Médecins Sans Frontières have provided integrated HIV/tuberculosis care in the rural Shiselweni region (210,000 population), which is served by 23 primary health clinics and three secondary health facilities. In 2012, routine VL monitoring was introduced, serving an estimated ART cohort of 15,000 patients. The HIV programme has been described elsewhere 23. Briefly, the national treatment guidelines 27 follow WHO recommendations, with routine VL testing performed at six and twelve months after ART initiation, and annually thereafter. A biocentric generic HIV VL platform is used for VL testing. In the case of an elevated VL, an enhanced adherence intervention is triggered to assess and rectify adherence barriers, with the first counselling session occurring at the clinic visit following the elevated VL. Patients receiving a VL test receive a month's pill refill, with patients with an elevated VL remaining on a monthly pill refill schedule to facilitate EAC. Patients receive three consecutive counselling sessions over a period of three months facilitated by trained lay counsellors with involvement of nurses and psychologists for complicated cases. These sessions are recorded in a dedicated EAC register and within the patient file. If the subsequent VL is re‐suppressed, patients continue with the same treatment regimen. If the VL remains elevated, medical doctors switch to a second‐line regimen.

### 2.2 Study population

This retrospective cohort analysis included patients on first‐ and second‐line ART who were aged 18 years or over and had an elevated VL > 1000 copies/mL 1, 27 in any of the 23 primary care clinics in the Shiselweni region (Swaziland), between January 2013 and June 2014, and were followed‐up through December 2015. Additionally, the DR cohort included patients from three secondary care facilities that served the same population.

### 2.3 Patient identification and data extraction

Patients with elevated VLs were identified through the laboratory‐based VL database. Clinical, counselling and demographic information were extracted from individual patient files and facility‐based registers.

### 2.4 HIV DR testing and interpretation

Dried blood spots (DBS) were obtained consecutively from patients with virological failure in the same primary care clinics and from three secondary care facilities between August 2013 and September 2014, and sent to the Centers for Disease Control (Atlanta) for HIV DR testing 28.

DR mutations were analysed according to the HIVdb Stanford Genotypic Resistance Interpretation Algorithm, and DR was defined as low, intermediate or high resistance against the most commonly used antiretroviral drugs: nucleoside reverse transcriptase inhibitors (NRTIs) – zidovudine, lamivudine (3TC), abacavir (ABC), stavudine, didanosine and tenofovir (TDF); non‐nucleoside reverse transcriptase inhibitors (NNRTIs) – efavirenz (EFV) and nevirapine (NVP); and protease inhibitor (PI): ritonavir‐boosted lopinavir (LPV/r). DR requiring treatment switching was defined as any DR to both EFV and NVP for a patient on first‐line therapy and any LPV/r resistance for a patient on second‐line therapy. Owing to a long turnaround time in receiving the genotype results, the treatment switching decision was based on documented virological failure.

### 2.5 Variables and definitions

An elevated VL was defined as a VL > 1000 copies/mL 1, 27, virological failure as two consecutive elevated VLs measurements three months apart, and re‐suppression as a consecutive VL ≤ 1000 copies/mL following an elevated VL.

Patients were followed‐up from the time of first recorded elevated VL until uptake of the second consecutive VL test and from the time of the second elevated VL test to treatment switching in time‐to‐event analysis. Censoring occurred at the date of last clinic visit for patients lost to follow‐up (LTFU) at the facility (three months without a recorded clinic visit) or at the end of the observation period on 31 December 2015.

EAC was assumed to have been done if the adherence session was recorded in the patient's file or in the facility‐based EAC register.

### 2.6 Statistical analysis

We calculated counts and proportions of patients at every level of the cascade. A Pearson's chi‐square test was used to compare categorical variables, and the Kruskal–Wallis test was used for comparison of continuous variables. We used Kaplan–Meier survival curves to estimate cumulative probabilities of receiving a follow‐up VL test. LTFU was treated as a competing risk for switching to second‐line therapy after two consecutive elevated VLs. A Fine and Gray proportional subdistribution hazards model 29 was used to determine the factors associated with treatment switching in the presence of LTFU as a competing risk. We used logistic regression to determine the factors associated with VL re‐suppression following an elevated VL, using variables that had been shown to be associated with re‐suppression in previous studies 30, 31, 32, 33, 34, 35, 36. A parsimonious model was achieved using Wald tests and likelihood ratio tests. For patients who were LTFU and did not have a second VL result, we performed a sensitivity analysis looking at two extreme cases (where all re‐suppressed or none re‐suppressed) to assess the robustness of our findings. We also used a logistic regression model for the odds of LTFU versus completion of the study.

DR was determined for each individual first‐ and second‐line drug. Patients on first‐line therapy were considered eligible for switching (according to the genotype resistance testing results) if they had developed high‐, intermediate‐ or low‐level resistance to both NNRTIs (NVP and EFV), as were those on second‐line therapy if they had developed resistance to LPV/r. We calculated the positive predictive value (PPV) of the WHO treatment‐switching algorithm by using the number of people with genuine resistance requiring treatment switching over the total number of patients who received a genotype.

Statistical analysis was performed with STATA 14.1 37.

### 2.7 Ethics

Ethics approval was obtained from the MSF Ethics Review Board and the Scientific and Ethics Committee of the Ministry of Health of Swaziland.

## 3 Results and Discussion

### 3.1 Baseline characteristics

A total of 828 patients (Table 1) had at least one elevated VL recorded between January 2013 and June 2014. The median age was 35 years (interquartile range (IQR): 29, 44), and 542 (65.5%) patients were female. The median time on ART was 2.95 (IQR: 1.81, 4.31) years, 820 (99.0%) patients were on first‐line ART and the most common first‐line regimen was TDF/3TC/EFV (n = 330; 39.9%).

**Table 1:** Baseline characteristics of patients with an elevated VL who received a follow‐up VL, their VL outcomes and factors associated with VL re‐suppression (<1000 copies/mL) at second VL test done

	First VL elevated (VL > 1000 copies/mL), n (%)	Follow‐up VL done, n (%)	VL suppressed (<1000 copies/mL), n (%)	cOR (95% CI)	*p* value	aOR (95% CI) (n = 695)	*p* value
Total	828	696 (84.1%)	410 (49.5%)				
Sex							
Female	542 (65.5)	451 (64.8)	265 (64.6)	Reference	__		
Male	286 (34.5)	245 (35.2)	145 (35.4)	1.02 (0.74 to 1.40)	0.913		
Baseline regimen (n = 703)							
AZT/3TC/NVP	222 (26.8)	194 (27.9)	103 (25.1)	Reference	__		
AZT/3TC/EFV	58 (7)	51 (7.3)	31 (7.6)	1.37 (0.73 to 2.57)	0.327		
TDF/3TC/NVP	14 (1.7)	12 (1.7)	7 (1.7)	1.24 (0.38 to 4.03)	0.724		
TDF/3TC/EFV	330 (39.9)	275 (39.5	169 (41.2)	1.41 (0.97 to 2.04)	0.071		
D4T/3TC/NVP	58 (7)	51 (7.3)	31 (7.6)	1.37 (0.73 to 2.57)	0.327		
D4T/3TC/EFV	18 (2.2)	17 (2.4)	8 (1.9)	0.79 (0.29 to 2.12)	0.633		
ABC/3TC/NVP	1 (0.1)	1 (0.1)	0 (0)	__	__		
ABC/3TC/EFV	2 (0.2)	2 (0.3)	2 (0.5)	__	__		
Missing	125 (15.1)	93 (13.4)	59 (14.4)	__	__		
Age							
18 to 34	398 (48.1)	312 (44.8)	178 (43.4)	Reference	__	Reference	__
35 to 49	304 (36.7)	276 (39.7)	157 (38.3)	0.99 (0.72 to 1.38)	0.967	1.01 (0.72 to 1.40)	0.964
50 to 64	94 (11.4)	82 (11.8)	59 (14.4)	1.93 (1.14 to 3.29)	0.015	1.98 (1.16 to 3.40)	0.012
≥65	30 (3.6)	25 (3.6)	16 (3.9)	1.34 (0.57 to 3.12)	0.5	1.32 (0.56 to 3.13)	0.52
Missing	2 (0.2)	1 (0.1)	0 (0)	__	__	__	__
Health zone							
Nhlangano	270 (32.6)	209 (30.0)	119 (29)	Reference	__		
Hlathikhulu	288 (34.8)	259 (37.2)	161 (39.3)	1.24 (0.86 to 1.80)	0.252		
Matsanjeni	270 (32.6)	228 (32.8)	130 (31.7)	1.00 (0.69 to 1.47)	0.987		
EAC							
Zero sessions	252 (30.4)	164 (23.6)	89 (21.7)	Reference	__	Reference	__
One session	133 (16.1)	116 (16.7)	68 (16.6)	1.19 (0.74 to 1.93)	0.47	1.28 (0.78 to 2.10)	0.319
Two sessions	155 (18.7)	144 (20.7)	86 (21)	1.25 (0.79 to 1.97)	0.335	1.40 (0.88 to 2.23)	0.152
Three sessions	288 (34.8)	272 (39.1)	167 (40.7)	1.34 (0.91 to 1.98)	0.143	1.51 (1.01 to 2.26)	0.045
Treatment regimen							
First line	820 (99)	653 (93.8)	375 (91.5)	Reference	__	Reference	__
Second line	8 (1)	43 (6.2)	35 (8.5)	2.87 (1.36 to 6.07)	0.006	3.53 (1.59 to 7.83)	0.002
Treatment outcome							
Retained in care	582 (70.3)	565 (81.2)	355 (86.6)	Reference	__		
LTFU	246 (29.7)	131 (18.8)	55 (13.4)	0.43 (0.29 to 0.63)	<0.001		
Time on ART	2.95 years (IQR: 1.81, 4.31)	2.96 years (IQR: 1.86, 4.32)	2.66 years (IQR: 1.73, 4.02)	1 (0.99 to 1.00)	0.161		

ABC, abacavir; aOR, adjusted odds ratio; ART, antiretroviral therapy; AZT, zidovudine; cOR, crude odds ratio; D4T, stavudine; EAC, enhanced adherence counselling; EFV, efavirenz; IQR, interquartile range; LTFU, lost to follow‐up; NRTI, nucleoside reverse transcriptase inhibitors; NVP, nevirapine; TDF, tenofovir; VL, viral load.

### 3.2 Lost to follow‐up

Of the 828 patients, 246 (29.7%) were LTFU. The proportion of patients LTFU differed by health zone (*p *=* *0.007), were younger (*p *< 0.001), more likely to be on first‐line (*p *< 0.001) and less likely to have received any EAC (*p *< 0.001) but did not differ by sex or drug regimen. In the multivariable logistic regression, only EAC remained associated with LTFU.

### 3.3 Enhanced adherence counselling

Of the 828 patients, 252 (30.4%) did not receive EAC, 133 (16.1%) received one session, 155 (18.7%) received two sessions and 288 (34.8%) received all three sessions. The median time from a first elevated VL to the first, second and third EAC session was 42 (IQR: 28, 73), 83 (IQR: 60, 114) and 113 (IQR: 91, 147.5) days respectively.

In our cohort, patients who received EAC were more likely to re‐suppress VL, with an increased likelihood of re‐suppression with each additional EAC session. In total, 84.1% of patients with a first elevated VL received a follow‐up VL of whom 58.9% re‐suppressed.

### 3.4 VL re‐suppression

Of the 828 patients, 696 (84.1%) received a follow‐up VL test by the end of the observation period. Of the 132 who did not have a follow‐up VL, 115 (87.1%) were LTFU, and 17 (12.9%) were retained on ART. The median time to the follow‐up VL test was 4.6 months (IQR: 3.3, 8.9), and VL test uptake at three and six months were 15.6% (95% CI: 13.3 to 18.3) and 62.1% (95% CI: 58.8 to 65.4).

Compared with findings from the early rollout in this setting 22, 23, follow‐up VL testing increased to 84% (vs. 70%) and VL re‐suppression remained similar (59% vs. 62%).

Overall, 410 (58.9%) of the 696 patients with a follow‐up VL had re‐suppressed: 321 (60.3%) of 532 patients who received at least one EAC and 89 (54.3%) of 164 patients without EAC. In multivariable logistic regression (Table 1), positive predictors of re‐suppression were age 50 to 64 (aOR: 1.98; *p *=* *0.012) compared with age 18 to 34 years and being on second‐line treatment (aOR: 3.53; *p *=* *0.002). The odds of re‐suppression increased with each consecutive EAC compared with patients receiving no intervention, being 1.28 (*p *=* *0.319), 1.40 (*p *=* *0.152) and 1.51 (*p *=* *0.045) for one, two and three EAC sessions. Only second‐line treatment remained significantly associated following sensitivity analysis.

This analysis indicated an increase in VL re‐suppression with each additional EAC session received compared with not receiving counselling. This finding was in contrast to the early rollout, in which EAC did not appear to have an additional benefit 22. EAC may have become more efficient during the scale‐up of VL testing in terms of messaging, patient support and intensity with proportionally more patients receiving all three EAC sessions.

Age between 50 and 64 years, being on a second‐line regimen and number of EAC sessions were also positive predictors of re‐suppression. Older age has been shown to be a predictor of re‐suppression in other studies 22, 36 and LPV/r‐based second‐line regimen are known to be more robust 38 as also confirmed by the genotyping results. Patients who received EAC sessions were more likely to re‐suppress, and this showed a dose–response relationship demonstrating the merits of this step in the VL cascade which corresponds with other studies’ findings 39, 40.

### 3.5 Treatment switching

Among 278 (39.9%) patients eligible for treatment switching, only 43.2% had their first‐line treatment switched.

Of the 696 patients with a follow‐up VL, 286 (41.1%) had presumptive virological failure. Eight (2.8%) were already on second‐line treatment before the VL result was available, leaving 278 (97.2%) eligible to be switched to a second‐line regimen after virological failure. Overall, 120 (43.2%) had switched by the end of the observation period, and the median time to treatment switching was 5.9 months (IQR: 2.3, 9.9) from the date the result was received back at the facility. Kaplan–Meier estimates showed that 12.6% (95% CI: 9.2 to 17.2) had switched regimens at three months after the date of blood sampling and 22.1% (95% CI: 17.6 to 27.4) had switched at six months.

Thirty‐five patients were switched to second‐line after a single VL > 1000 copies/mL (median time since VL 35 days, IQR: 24, 63). A review of their VL history showed that 17 patients had an elevated VL at their most recent previous VL test.

In multivariable competing risk regression (Table 2), an ABC‐based treatment regimen (aHR: 8.58; *p *< 0.001) was associated with increased probability of treatment switching, but only one patient was in this group. Receiving one, two or three EAC sessions showed a tendency towards increased treatment switching, but this was not statistically significant.

**Table 2:** Baseline characteristics of patients eligible for a treatment switch, their treatment outcomes and factors associated with treatment switching

	Eligible to be switched to second line (second VL > 1000 copies/mL), n (%)	Switched to second line, n (%)	cSHR (95% CI)	*p* value	aSHR (n = 219) (95% CI)	*p* value
Total	278	120 (43.2)				
Sex						
Female	179 (64.6)	78 (65)	Reference	__		
Male	99 (35.4)	42 (35)	0.95 (0.65 to 1.39)	0.809		
Baseline regimen						
AZT/3TC/NVP	88 (31.7)	40 (33.3)	Reference	__	Reference	__
AZT/3TC/EFV	20 (7.2)	8 (6.7)	0.90 (0.43 to 1.91)	0.795	0.94 (0.44 to 1.99)	0.878
TDF/3TC/NVP	5 (1.8)	1 (0.8)	0.36 (0.05 to 2.49)	0.301	0.36 (0.06 to 2.27)	0.278
TDF/3TC/EFV	104 (37.4)	46 (38.3)	0.98 (0.64 to 1.48)	0.913	0.97 (0.63 to 1.50)	0.909
D4T/3TC/NVP	17 (6.1)	5 (4.2)	0.71 (0.28 to 1.78)	0.465	0.76 (0.30 to 1.98)	0.581
D4T/3TC/EFV	9 (3.2)	4 (3.3)	1.20 (0.40 to 3.54)	0.746	1.05 (0.33 to 3.26)	0.937
ABC/3TC/NVP	1 (0.4)	1 (0.8)	6.69 (4.44 to 10.06)	<0.001	8.58 (4.82 to 15.26)	<0.001
Missing	34 (12.2)	15 (12.5)	__			
Age						
18 to 34	130 (46.9)	58 (48.7)	Reference	__		
35 to 49	116 (41.5)	50 (41.2)	0.91 (0.63 to 1.34)	0.649		
50 to 64	22 (7.9)	9 (7.6)	0.78 (0.39 to 1.54)	0.472		
≥65	9 (3.3)	3 (2.5)	0.50 (0.16 to 1.52)	0.222		
Missing	1 (0.4)	0 (0)	__			
Health zone						
Nhlangano	88 (31.8)	38 (31.9)	Reference	__		
Hlathikhulu	95 (34.3)	42 (35.3)	1.46 (0.95 to 2.23)	0.081		
Matsanjeni	95 (33.9)	40 (32.8)	1.30 (0.83 to 2.04)	0.243		
EAC						
Zero sessions	72 (25.6)	26 (21.0)	Reference	__	Reference	__
One session	44 (15.9)	21 (17.7)	1.25 (0.71 to 2.18)	0.436	1.22 (0.66 to 2.23)	0.523
Two sessions	58 (20.9)	26 (21.8)	1.62 (0.95 to 2.76)	0.077	1.69 (0.93 to 3.09)	0.087
Three sessions	104 (37.6)	47 (39.5)	1.31 (0.82 to 2.10)	0.263	1.30 (0.77 to 2.19)	0.322
Treatment outcome						
Retained in care	202 (72.6)	120 (100)				
LTFU	76 (27.4)	0 (0)				
Genotype						
No genotype	205 (73.6)	77 (63.9)				
Genotype done	73 (26.4)	43 (36.1)				
Successful genotype						
No result	220 (79.1)	83 (68.9)				
Genotype result available	58 (20.9)	37 (31.1)				
Time on ART	3.15 years (IQR: 1.95, 4.69)	3.26 years (IQR: 2.27, 4.79)	1 (0.99 to 1.00)	0.181		

ABC, abacavir; ART, antiretroviral therapy; aSHR, adjusted subdistribution hazard ratio; AZT, zidovudine; cSHR, crude subdistribution hazard ratio; D4T, stavudine; EAC, enhanced adherence counselling; EFV, efavirenz; IQR, interquartile range; LTFU, lost to follow‐up; NRTI, nucleoside reverse transcriptase inhibitors; NVP, nevirapine; TDF, tenofovir; VL, viral load.

Treatment switching remained low (43.0% at the end of follow‐up), higher compared with the early rollout 23 and comparable to other studies 41, 42. In this setting, clinicians are instructed to switch at time of virological failure but delayed treatment switching. Without irrefutable evidence of the ineffectiveness of the current treatment, clinicians may aim for conservation of treatment options when therapeutic options are limited 30, 43.

**Figure 1:**
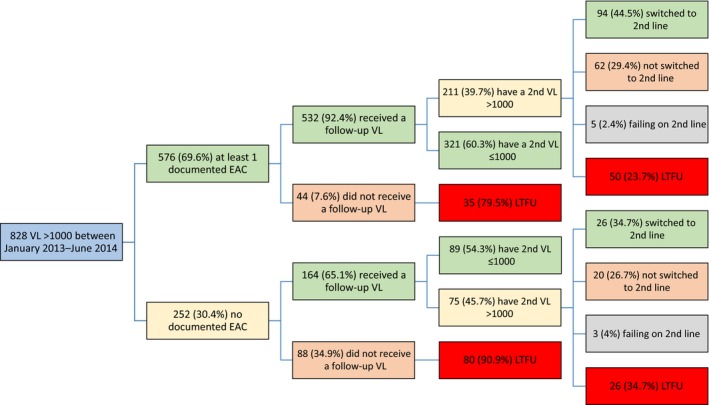
The viral load and treatment switching cascade. EAC, enhanced adherence counselling; LFTU, lost to follow‐up; VL, viral load.

The cascade is illustrated in Figure 1. The disengagement from care between the first and second VL tests and between the second elevated VL and treatment switching is of concern as it may contribute to increased mortality and onward transmission 44, 45, 46.

### 3.6 HIV drug resistance testing

In total, 214 DBS samples were collected from adults with virological failure in primary and secondary care facilities. Of these, 155 (72.4%) were successfully sequenced, with 144 (92.9%) patients on first‐line ART, 10 (6.5%) on second‐line ART and 1 (0.6%) missing regimen information. Ninety‐three (60.0%) patients were female, sixty (38.7%) were male and two (1.3%) had missing sex information. Sixty‐seven (43.2%) patients were from primary care clinics, seventy‐nine (51.0%) were from secondary care facilities and nine (5.8%) had missing health facility information. Resistance to both EFV and NVP ranged from 87.5% to 100% by regimen (*p *=* *0.881), and resistance to LPV/r ranged from 0% to 66.7% by regimen (*p *=* *0.054) (Table 3).

**Table 3:** DR by antiretroviral therapy regimen

	First line, n (%)							*p* value	Second line, n (%)			*p* value
Regimen	AZT/3TC/NVP	AZT/3TC/EFV	TDF/3TC/NVP	TDF/3TC/EFV	D4T/3TC/NVP	D4T/3TC/EFV	ABC/3TC/EFV		ABC/DDI/LPV/r	AZT/3TC/LPV/r	TDF/3TC/LPV/r	
Total	59	26	13	40	3	2	1	144	3	3	4	10
AZT	44 (74.6)	13 (50.0)	5 (38.5)	7 (17.5)	2 (66.7)	1 (50)	0 (0)	<0.001	1 (33.3)	0 (0)	2 (50.0)	0.356
3TC	55 (93.2)	23 (88.5)	12 (92.3)	34 (85)	3 (100)	2 (100)	1 (100)	0.851	2 (66.7)	2 (66.7)	3 (75.0)	0.961
TDF	29 (49.2)	12 (46.2)	7 (53.8)	31 (77.5)	1 (33.3)	2 (100)	0 (0)	0.041	2 (66.7)	2 (66.7)	1 (25.0)	0.435
ABC	55 (93.2)	23 (88.5)	12 (92.3)	34 (85)	3 (100)	2 (100)	1 (100)	0.851	2 (66.7)	2 (66.7)	3 (75.0)	0.961
D4T	42 (71.2)	15 (57.7)	9 (69.2)	31 (77.5)	2 (66.7)	2 (100)	0 (0)	0.400	1 (33.3)	1 (33.3)	2 (50.0)	0.87
DDI	44 (74.6)	18 (69.2)	9 (69.2)	33 (82.5)	2 (66.7)	2 (100)	1 (100)	0.805	2 (66.7)	2 (66.7)	2 (50.0)	0.87
NVP	56 (94.9)	24 (92.3)	12 (92.3)	35 (87.5)	3 (100)	2 (100)	1 (100)	0.881	2 (66.7)	2 (66.7)	3 (75.0)	0.961
EFV	56 (94.9)	24 (92.3)	12 (92.3)	35 (87.5)	3 (100)	2 (100)	1 (100)	0.881	2 (66.7)	2 (66.7)	3 (75.0)	0.961
LPV/r	1 (1.7)	0 (0)	0 (0)	0 (0)	0 (0)	0 (0)	0 (0)	0.963	2 (66.7)	0 (0)	0 (0)	0.054

3TC, lamivudine; ABC, abacavir; AZT, zidovudine; D4T, stavudine; DDI, didanosine; DR, drug resistance; EFV, efavirenz; LPV/r, ritonavir‐boosted lopinavir; NVP, nevirapine; TDF, tenofovir.

The WHO algorithm accurately predicted resistance requiring a switch to second‐line therapy. Of the 144 first‐line patients, 133 (92.4%) had low, intermediate or high resistance to both EFV and NVP and were eligible for switching to second‐line ART (PPV for the need for treatment switching). This equated to 61 (95.3%) of 64 patients from primary health clinics and 63 (88.7%) of 71 patients from secondary health facilities. Only two (20%) of the second‐line patients had resistance to LPV/r.

The WHO algorithm had high accuracy for predicting failure of first‐line ART as also reported from other sub‐Saharan settings, where resistance ranged from 53% to 93% 47, 48, 49, 50. Following a public health approach, patients failing first‐line regimens do not need DR testing for confirmation of treatment failure before treatment switching. This algorithm, however, showed a low PPV for patients on second‐line therapy (20%), therefore requiring HIV DR testing to avoid premature switches to third‐line ART. Other studies also showed that a minority of patients with virological failure had PI DR levels requiring treatment switching 39, 51.

This study had several limitations. The use of routine data is prone to suboptimal data quality and completeness, and leakages in our cascade might bias our findings. We could not ascertain patients’ final outcome and differentiate between patients LTFU and deceased. Because of the small number of second‐line patients, any significant associations for this group should be interpreted with caution.

## 4 Conclusions

VL monitoring has the potential to improve ART programmes and to bolster treatment support. We showed that the WHO VL algorithm works well for patients on first‐line ART. However, the ramifications of any delay are serious given the number of patients LTFU between each cascade step. Finally, for patients with suspected virological failure, even in the face of ongoing adherence problems, treatment switching should be considered as our data show that they most likely do have resistant mutations.

## Competing interests

The authors declare that they have no competing interests.

## Authors’ contributions

KJ, RT, GM, IC, BK and SMK conceptualized, designed and developed the protocol. GM, MP, DE, IZ, MN, BK, CGE, CY and SMK implemented the research. MN, DE and CY collected the data. DE, MN and BK statistically analysed the data. BK, DE, IC, MN, RT, SMK, JG and RT interpreted the findings of the data. DE, BK and IC wrote the first draft of the manuscript. All authors read and approved the final manuscript.
